# Effect of Root Dentin Pretreatment on Micro-Push-Out Bond Strength of Fiber Posts to Root Canal Dentin: Cold Atmospheric Argon Plasma (CAAP) and Ethylenediaminetetraacetic Acid (EDTA)

**DOI:** 10.1155/2021/5571480

**Published:** 2021-05-28

**Authors:** Farzaneh Sadeghi Mahounak, Mahdi Abbasi, Naghmeh Meraji, Maryam Rezazadeh Sefideh, Mohammad Javad Kharrazi Fard, Elham Ahmadi, Ladan Ranjbar Omrani

**Affiliations:** ^1^Department of Restorative Dentistry, Shahid Beheshti University of Medical Science, Tehran, Iran; ^2^Department of Restorative Dentistry, Tehran University of Medical Science, Tehran, Iran; ^3^Private Practice, Tehran, Iran; ^4^Department of Endodontics, Tehran University of Medical Science, Tehran, Iran; ^5^Tehran University of Medical Science, Tehran, Iran; ^6^Dental Research Center, Dentistry Research Institute, Tehran University of Medical Science, Tehran, Iran

## Abstract

**Purpose:**

Debonding from the root canal dentin is the most common failure mode of fiber posts. This study aimed to assess the effects of cold atmospheric argon plasma (CAAP) and ethylenediaminetetraacetic acid (EDTA) on micro-push-out bond strength of fiber posts to root canal dentin.

**Materials and Methods:**

Forty maxillary canine teeth were decoronated, underwent endodontic treatment, and were stored in an incubator for 7 days. After post space preparation, the teeth were randomly divided into four groups for different surface treatments: (I) saline, (II) 17% EDTA, (III) CAAP, and (IV) 17% EDTA + CAAP. Fiber posts (Whitepost no. 2, FGM) were cemented into the root canals using Panavia F2.0 resin cement, and 1 mm-thick sections were made at the coronal, middle, and apical thirds of the roots. The samples underwent micro-push-out bond strength test. The mode of failure was also determined under a stereomicroscope. Data were analyzed using three-way ANOVA and Tukey's post hoc test (*α* = 0.05). The mode of failure data were analyzed using the chi-square test.

**Results:**

The mean micro-push-out bond strength of fiber posts was not significantly different in the four groups (*P* > 0.05). However, the bond strength values in the coronal third were significantly higher than the corresponding values in the apical third (*P*=0.01). There was no significant difference in the modes of failure between the groups (*P* > 0.05).

**Conclusion:**

Application of CAAP alone or in combination with 17% EDTA could not successfully increase the bond strength of fiber posts to root canal dentin.

## 1. Introduction

After endodontic treatment, the shape and function of the tooth crown are often restored by post-retained restorations, depending on the amount of the lost tooth structure [1]. Prefabricated posts are widely used in dentistry due to their placement in one single session and easy application. Fiber posts have advantages such as a modulus of elasticity comparable to that of the tooth structure and appropriate stress distribution [2].

Several clinical studies have reported that debonding of intracanal posts is the most common type of failure of restorations retained by fiber-reinforced posts [3, 4]. Since fiber posts are passively retained in the root canal, adhesive resin cements and the bonding process play a fundamental role in the long-term clinical service of these restorations [5].

Resin cements are classified into total-etch, self-etch, and self-adhesive cements. The total-etch systems create a high bond strength; however, they are technique sensitive and have a risk of overetching of dentin. The problems related to total-etch cements may be minimized by the use of self-etch systems that cause lower decalcification and overetching of dentin as the result of the application of weaker acids [6]. However, some problems such as a thicker smear layer [7], poor moisture control [8], low acidity [9], and neutralization of functional acidic monomers of self-etch primers [10] in root dentin question the quality of penetration of self-etch adhesives into root dentin. To overcome the limitations of adhesives in the root canal system, some attempts were recently made to develop dentin surface modification techniques and some chemical and electrical methods to enhance the penetration and absorption of bonding agents.

Ethylenediaminetetraacetic acid (EDTA) is a cationic agent used for chelation of calcium ions, removing the inorganic elements, collagen rearrangement, and elimination of the smear layer in the bonding process of restorative materials [11, 12]. Treatment of coronal dentin with EDTA prior to the application of self-etch primers increases the bond strength and creates a homogenous, thin hybrid layer [11, 13].

Cold atmospheric argon plasma (CAAP) is an effective surface modification method that has recently gained the spotlight [14–16]. In this technique, high-energy electrons, ionic particles, and free radicals are degraded and interact with the surface of material and modify the superficial layer [16].

CAAP includes charged ions, radicals, and photons that can increase the surface energy and confer hydrophilic properties to the surface of solid materials by removing the hydrocarbon and offering hydroxyl groups [16]. This property plays a significant role in better penetration of adhesive. Also, surface treatment with plasma may chemically convert the organic materials on the dentin surface into volatile components and lead to the opening of dentinal tubules. Thus, it can be used as a method to eliminate the organic debris from the dentinal tubules [17]. Recent studies [18, 19] have shown that surface treatment with nonthermal plasma can enhance the bond strength of the composite to dentin. Some previous studies attempted to improve the bonding of sealers and resin cements by applying CAAP on root canal dentin [20, 21]. However, search of the literature yielded no study on its application in the post space considering the complexities of bonding fiber posts to root dentin. Stancompiano et al. [20] showed that EDTA removed the smear layer by its chelating effect and also demonstrated enhanced penetration and wettability obtained following the use of plasma. The present study aimed to assess the effect of CAAP alone and in combination with EDTA on the bond strength of tooth-colored posts to root dentin. The null hypothesis of this study was that CAAP alone or in combination with EDTA would not enhance the bond strength of fiber posts to root dentin.

## 2. Materials and Methods

The minimum sample size required for each of the four study groups was 10 samples according to a study by Garcia et al. [22] using the one-way ANOVA power analysis feature of PASS 11 software.

A total of 40 single-rooted maxillary canine teeth with a minimum root length of 14 mm that had been extracted due to periodontal reasons were used in this study (ethical approval code: IR.TUMS.DENTISTRY.REC.1397.128). The exclusion criteria were the presence of carious lesions or previous restorations, previous root canal treatment, and the presence of >25° root curvature or root cracks. The teeth were disinfected by immersion in 0.5% chloramine-T solution for 1 week. The teeth were stored in distilled water until the time of use (maximum of 3 months). The crowns were cut at 1 mm coronal to the cementoenamel junction using a diamond disc (Yuanda, China) under copious water irrigation. The root canals were then instrumented with S1 to F2 ProTaper rotary files (Dentsply, USA). A #10 K-file (Mani, Japan) was inserted into the root canal until its tip was visible at the apical foramen. The working length was determined by decreasing this length by 1 mm. The root canals were then instrumented with S1 to F2 ProTaper rotary files (Dentsply, USA). The irrigation protocol used during root canal instrumentation included the use of 5 mL of 5.25% sodium hypochlorite (Cerkamed, Poland) and a final flush with 5 mL of 17% EDTA (Cerkamed, Poland) followed by the use of 5 mL of 5.25% sodium hypochlorite. Next, the canals were rinsed with 10 mL of saline (Samen, Iran) and dried with paper points (Spident, Korea). The canals were filled with gutta-percha (Spident, Korea) and AH Plus resin sealer (Dentsply, USA) using the lateral compaction technique. The gutta-percha was cut 1 mm below the canal orifice, and the remaining space was temporarily filled with temporary restorative material (Cavit; Ariadent, Iran). The samples were incubated at 37°C in 100% humidity for 1 week (Thermo Scientific™ Heratherm™; Thermo Fisher Scientific Inc., USA).

Next, 10 mm of the coronal gutta-percha was removed using a #2 Peeso Reamer (Mani, Japan), and then a #2 drill, provided by the manufacturer (FGM, Brazil), was used for post space preparation. The teeth were randomly divided into four groups, and pretreatments were performed as follows:

  Group 1: 5 mL of 0.9% sodium chloride was injected into the canal space and remained for 1 min. The canals were then dried with paper points.
  Group 2: flushing with 5 mL of 17% EDTA was performed for 1 min. Then, the canals were washed with 5 mL of distilled water and dried with paper points.
  Group 3: an atmospheric plasma jet (MEDAiON; Nik Fanavaran Plasma, Iran) with a potential difference of 4.5 kV and a frequency of 80 kHz and argon gas with a flow rate of 3000 SCCM (3 L/min) were used for 1 min such that the nozzle head was positioned at a distance of 2 mm from the canal orifice [23].
  Group 4: flushing was performed with 5 mL of 17% EDTA for 1 min. After washing and drying, the atmospheric plasma jet with the same conditions mentioned for the third group was used for 1 min.

Immediately after the root dentin surface treatment, fiber posts (Whitepost no. 2, FGM, Brazil) were cement with Panavia F2.0 resin cement (Kuraray, Japan) according to the manufacturer's instructions. After proper mixing of ED primers A and B for 5 s, the mixture was applied into the post space prepared by using the drill recommended by the manufacturer. Paper points were used to remove the excess primer. The two cement pastes were mixed and applied on the surface of the posts. The posts were placed in the prepared post spaces by finger pressure. After removal of excess cement with a cotton pellet, photoactivation was performed for 40 s (Woodpecker LED.B; Guilin Woodpecker Medical Instrument Co., Guilin, Guangxi, China) with an output power of 1000 mW/cm^2^. The samples were then immersed in distilled water for 24 h. Next, they were mounted in metal molds using transparent polyester.

A diamond disc under the air coolant (Mecatome; Presi, France) was used to obtain 1 mm-thick sections from the coronal, middle, and apical thirds of the roots. The thickness of slices was checked using a digital caliper (Mitutoyo, Japan) with 0.01 mm accuracy. The coronal side of each slice was marked by a waterproof marker.

The micro-push-out bond strength was then measured using a universal testing machine (ZwickRoell, Germany). For this purpose, the specimens were placed on a special acrylic jig such that their apical surface faced up. A plunger with 0.8 mm diameter applied load to the center of the posts at a crosshead speed of 1 mm/min. The maximum load tolerated by the posts was recorded in Newton (N), and the bond strength value was calculated using the following formula:

(1) $$\begin{array}{c} \text{micro}-\text{push}-\text{out bond strength}=\frac{F}{A}\left[A=\pi \left(R1+R2\right)\nu \left\{\left(R1-R2\right)2+H2\right\}\right]. \end{array}$$

*R*1 is the radius of the post space at the coronal aspect of the specimen, *R*2 is the radius of the post space at the apical aspect of the specimen, and *H* is the height (thickness) of the slice.

The specimens were then inspected under a stereomicroscope (Nikon, Japan) at ×10 magnification to assess the mode of failure, which was classified as follows:

  AD: adhesive failure between dentin and cement (no cement remaining on the post)
  AP: adhesive failure between cement and post (post is entirely covered with resin cement)
  CD: cohesive failure in dentin
  CP: cohesive failure in the post
  M1: mixed failure such that 0% to 50% of the post surface was covered with cement
  M2: mixed failure such that 50% to 100% of the post surface was covered with cement

Data were analyzed using SPSS via three-way ANOVA and post hoc Tukey's test to assess the effect of different root dentin surface treatments on bond strength of tooth-colored posts to root dentin in the apical, middle, and cervical thirds. The level of significance was set at 0.05. The mode of failure was analyzed using the chi-square test.

## 3. Results

Three-way ANOVA revealed no significant difference among the groups regarding the mean bond strength to root dentin (*P* > 0.05).Tukey's post hoc test revealed that the bond strength in the coronal third was significantly higher than that in the apical third (*P*=0.01), but the middle third had no significant difference with the coronal or apical third in this respect (*P* > 0.05).

**Figure 1:**
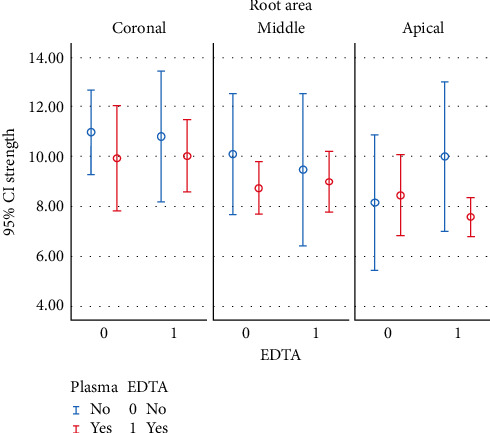
Bond strength in different parts of the root based on the type of surface treatment.

As shown in Figure 1, the maximum mean bond strength was noted in group 1 (control) in the coronal third, while the lowest mean bond strength was noted in group 3 (plasma) in the apical third.

Table 1 presents the frequency distribution of the modes of failure in different parts of the root. According to the chi-square test, the groups were not significantly different in this respect (*P* > 0.05).

## 4. Discussion

The micro-push-out test is highly similar to the clinical situation due to the homogenous stress distribution [24]. Thus, a micro-push-out test was used in this study to measure the bond strength of the fiber post to root dentin in the coronal, middle, and apical thirds. No significant difference existed in any part of the root between different surface treatment groups. The null hypothesis of the study was confirmed.

The bond strength in the coronal third was significantly higher than that in the apical third. Dentinal tubules are cleaner, larger, denser, and more vertically oriented in the coronal region and provide a higher bond strength to the fiber post in this region. This finding was in accordance with the results of previous studies [25, 26]. Inadequate polymerization in the apical region seems to be another reason for this finding.

It appears that the application of 17% EDTA for surface treatment before the use of a self-etch adhesive system creates a thinner demineralized layer with nondenatured collagen fibrils, which has higher mineral content in its interfibrillar spaces. Creation of a stiffer collagen network and a higher number of hydroxyapatite crystals in EDTA-demineralized dentin provide less shrinkage during the infiltration of resin copolymers [27]. Such a lower polymerization stress appears to have a less distractive effect on bond strength; however, the results of the present study did not confirm this theory. Our finding was in line with the results of previous studies by Barreto et al. [28] and Garcia et al. [22]. Some studies have shown that increasing the time of exposure and concentration of EDTA can remove more calcium ions from dentin and create a nonmineral area close to the surface [28, 29]. This process may be destructive for the bond strength of self-adhesive resin cements to root dentin due to fewer chemical interactions between the acidic monomers of this type of cement and calcium ions [22]. Also, evidence shows that dentin has a flat and smooth surface with regular distinct porosities following root canal irrigation with EDTA with concentrations higher than 15%, which is not optimal for adhesion [22]. The lower mean bond strength in the EDTA group in our study was in contrast to the results of Gu et al. [30]. This controversy could be due to the difference in the methodology since they filled the entire canal space with Panavia F2.0 resin cement without the fiber post.

It appears that CAAP can induce chemical bonding between HEMA and dentin collagen [31]. Moreover, the reactive oxygen species in the plasma can be used in the form of carbonyl groups in type I collagen molecules via surface interactions and are believed to play a role in increasing the hydrogen bonds between the collagen fibrils and adhesive [32]. Strong chemical and physical bonds formed by plasma treatment subsequently promote the migration of HEMA into demineralized dentin [18]. Although these mechanisms can be effective for better resin penetration into root dentinal tubules, this was not the case in our study.

Few studies have evaluated the effect of CAAP treatment of root dentin on bond strength of resin cements [20, 21]. Prado et al. found that argon plasma treatment increased the penetration of the resin sealer into the root canal dentin in the coronal third [33].

Type of plasma, duration of application, and the distance of the nozzle of the plasma jet from the treated surface affect the efficacy of plasma in resin bond strength. The effective plasma treatment time based on the data obtained from mechanical testing is 30 s, and longer durations (100 s and 300 s) decrease the bond strength because long-term plasma treatment can degrade the collagen fibers and weaken the interface [34]. In this study, plasma was used for 60 s due to the limited root canal space, difficult access of plasma to the entire length of the root canal, and also in order to benefit more from the advantages of plasma application. However, it appears that the time duration selected in this study was insufficient since other studies used plasma for 180 s and reported its optimal efficacy for the enhancement of resin cement bond strength [20, 21].

The distance between the nozzle of the plasma jet and the canal orifice was 2 mm in our study, which decreases the plasma penetration into the root canal system. This limited penetration depth of plasma in the root canal system can be one possible reason for not achieving the desired outcome in this study.

Moreover, the rewetting step with water after plasma treatment plays an essential role in partial opening of dentinal tubules and removal of the smear layer because the plasma gas consists of excited atomic and ionic species that act like molecular sandblasting. Without the rewetting process, the dislodged contaminants at atmospheric pressure may remain and redeposit or recombine on the dentin surface, but rewetting followed by air blowing can remove the plasma dislodged contaminants by excess water [35]. However, since the presence of excess water can impair the function of self-etch adhesives, we did not rehydrate the surface, which may be another reason for insignificant effect of plasma on bond strength.

Subsequent use of an organic solvent such as sodium hypochlorite can enhance further exposure of inorganic materials in the decalcified dentin substrate to EDTA following removal of the organic matrix [36, 37].

In the present study, adhesive failure of the post was more commonly observed in the control and EDTA groups. Also, cohesive failure of the post in the plasma group and cohesive failure of dentin in the EDTA + plasma group were dominant (Table 1). However, no similar study is available to compare our results with, and we found no significant difference in this respect between the groups.

Considering the novelty of the use of plasma technology in restorative dentistry and lack of knowledge about the efficient conditions to enhance dentin bonding, it appears that this technology still has a long way to go, and further studies with other plasma types and different working conditions and application times are required in order to be able to cast a final judgment regarding the potential of the plasma jet for post space treatment.

The post and root canal geometry [38, 39], type of resin cement [40], and mechanical and thermal cycling may affect the bond strength of the fiber post to root dentin. In this study, only one type of post and one type of cement without mechanical or thermal cycles were assessed. Thus, caution should be exercised in generalizing the results of this study to the clinical setting. Future studies with follow-up experiments are required to assess the bond strength of the fiber post in simulated oral conditions. Within the limitations of this study, it appears that application of CAAP for 1 min with or without 17% EDTA in the root canal system is not successful for the enhancement of bond strength.

### 4.1. Clinical Relevance

Cold atmospheric plasma with or without EDTA as a chelating agent could not improve the bond strength of the fiber post to root canal dentin.

## Figures and Tables

**Table 1:** Frequency distribution of the modes of failure in different parts of the root.

Failure mode	AD^1^	AP^2^	CD^3^	CP^4^	M1^5^	M2^6^
Apical third	6	13	11	2	4	4
Middle third	1	8	11	8	8	4
Coronal third	0	6	12	12	6	4

^1^Adhesive failure between dentin and cement (no cement remaining on the post). ^2^Adhesive failure between cement and post (post is entirely covered with resin cement). ^3^Cohesive failure in dentin. ^4^Cohesive failure in the post. ^5^Mixed failure such that 0% to 50% of the post surface was covered with cement. ^6^Mixed failure such that 50% to 100% of the post surface was covered with cement.

## Data Availability

The data used to support the findings of this study are available from the corresponding author upon request.
